# Supplementary material for: Oral Polio Vaccine Campaigns May Reduce the Risk of Death from Respiratory Infections
Source: Vaccines (Basel). 2021 Oct 4;9(10):1133. doi: 10.3390/vaccines9101133 (PMC8537441; doi:10.3390/vaccines9101133)

Supplementary Table S1. Diagnoses for cause of deaths, number of deaths and defined categories categories. Note that all deaths before 29 days of age are categorised as neonatal deaths.

**Cause of death**	**Number of deaths**	**Category**
Acute resp infect, incl pneumonia	127	Respiratory infection
Measles	4	Respiratory infection
Pertussis	2	Respiratory infection
Asthma	1	Respiratory infection
Meningitis and encephalitis	53	Other cause
Acute abdomen	17	Other cause
Diarrhoeal diseases	16	Other cause
HIV/AIDS related death	13	Other cause
Severe malnutrition	12	Other cause
Epilepsy	9	Other cause
Congenital malformation	7	Other cause
Sepsis (non-obstetric)	6	Other cause
Other and unspecified infect dis	5	Other cause
Other and unspecified cardiac dis	4	Other cause
Sickle cell with crisis	2	Other cause
Liver cirrhosis	2	Other cause
Renal failure	1	Other cause
Acute cardiac disease	1	Other cause
Other and unspecified neoplasms	1	Other cause
Neonatal pneumonia	48	Neonatal death
Neonatal sepsis	26	Neonatal death
Prematurity	16	Neonatal death
Other and unspecified neonatal Co	12	Neonatal death
Birth asphyxia	7	Neonatal death
Accid drowning and submersion	53	Accident
Other transport accident	11	Accident
Other and unspecified external Co	2	Accident
Road traffic accident	2	Accident
Accid expos to smoke, fire & flam	1	Accident
Contact with venomous plant/anima	1	Accident
Assault	1	Accident
Accid fall	1	Accident
Indeterminate	31	Missing cause

Supplementary Table S2. Deaths by cause of death, age group and timing of death in relation to oral polio vaccine campaigns among children within the Chakaria HDSS from 2012 to 2019.

	Number of deaths (rate per 100 person-years, person-years)						
	Neonatal period (<29 days)		Post-neonatal infant period (29 days-11 months)		Child period (12-35 months)		Total
	After-campaign	Before-campaign	After-campaign	Before-campaign	After-campaign	Before-campaign	
Neonatal deaths	1 (10.2, 10)	168 (17.0, 988)					169 (16.94, 998)
Respiratory infections			6 (0.31, 1907)	104 (0.86, 12147)	9 (0.08, 10613)	15 (0.09, 16086)	134 (0.33, 40753)
Other causes			10 (0.52, 1907)	59 (0.49, 12147)	14 (0.13, 10613)	20 (0.12, 16086)	103 (0.25, 40753)
Accidents			0 (0.00, 1907)	7 (0.06, 12147)	24 (0.23, 10613)	41 (0.25, 16086)	72 (0.18, 40753)
Missing cause			1 (0.05, 1907)	12 (0.10, 12147)	3 (0.03, 10613)	2 (0.01, 16086)	18 (0.04, 40753)

Supplementary Table S3. Mortality rates (per 100 person-years) and hazard ratios (HR) for after-campaign versus before-campaign for children only eligible to oral polio vaccine (OPV), by cause of death and age group. Analysis from 29 days to 35 months of age among children within the Chakaria HDSS from 2012 to 2019.

Respiratory infections

Campaign	Mortality rates per 100 person years (deaths / person years)		HR (After/Before-campaign) (95% CI) #1	Main model HR (After/Before-campaign) (95% CI) #2
	After-campaign	Before-campaign		
**29 days – 11 months**				
Campaign-OPV-only	0.31 (6/1907)	0.86 (104/12147)	0.45 (0.20-1.00)*	0.40 (0.18-0.91)*
Campaign-OPV+VAS	0.40 (4/1000)	0.81 (106/13055)	0.73 (0.27-1.95)	1.19 (0.45-3.17)
Campaign-OPV+MV	1.78 (1/56)	0.78 (109/13998)	4.95 (0.64-38.5)	8.54 (0.99-73.7)
Campaign-VAS-only	0.51 (15/2922)	0.85 (95/11133)	1.19 (0.62-2.26)	1.08 (0.57-2.05)
Campaign-MV-only	1.88 (1/53)	0.78 (109/14001)	18.0 (1.60-203)*	18.1 (1.60-205)*
**12 – 35 months**				
Campaign-OPV-only	0.08 (9/10613)	0.09 (15/16086)	0.58 (0.19-1.73)	0.32 (0.08-1.24)
Campaign-OPV+VAS	0.07 (3/4043)	0.09 (21/22656)	0.68 (0.20-2.27)	1.58 (0.44-5.64)
Campaign-OPV+MV	0.11 (5/4715)	0.09 (19/21984)	1.40 (0.40-4.98)	2.14 (0.51-8.92)
Campaign-VAS-only	0.08 (17/20047)	0.11 (7/6652)	0.96 (0.29-3.16)	0.92 (0.34-2.47)
Campaign-MV-only	0.04 (2/4777)	0.10 (22/21922)	0.55 (0.12-2.45)	0.51 (0.11-2.34)

<u>Other causes</u>

Campaign	Mortality rates per 100 person years (deaths / person years)		HR (After/Before-campaign) (95% CI) #1	Main model HR (After/Before-campaign) (95% CI) #2
	After-campaign	Before-campaign		
**29 days – 11 months**				
Campaign-OPV-only	0.52 (10/1907)	0.49 (59/12147)	1.18 (0.56-2.49)	1.40 (0.68-2.90)
Campaign-OPV+VAS	0.30 (3/1000)	0.51 (66/13055)	0.48 (0.15-1.50)	0.47 (0.15-1.50)
Campaign-OPV+MV	3.56 (2/56)	0.48 (67/13998)	13.8 (2.76-69.0)*	10.6 (1.93-58.5)*
Campaign-VAS-only	0.55 (16/2922)	0.48 (53/11133)	1.65 (0.84-3.24)	1.61 (0.83-3.09)
Campaign-MV-only	0.00 (0/53)	0.49 (69/14001)	-	-
**12 – 35 months**				
Campaign-OPV-only	0.13 (14/10613)	0.12 (20/16086)	0.62 (0.20-1.86)	0.82 (0.25-2.73)
Campaign-OPV+VAS	0.15 (6/4043)	0.12 (28/22656)	0.84 (0.30-2.39)	0.79 (0.20-3.10)
Campaign-OPV+MV	0.08 (4/4715)	0.14 (30/21984)	0.49 (0.14-1.67)	0.41 (0.11-1.47)
Campaign-VAS-only	0.13 (26/20047)	0.12 (8/6652)	1.03 (0.32-3.33)	0.94 (0.39-2.25)
Campaign-MV-only	0.13 (6/4777)	0.13 (28/21922)	1.68 (0.64-4.40)	1.58 (0.57-4.36)

#1: Adjusting for age (underlying time) and year*age group.
#2: Main multivariable model: adjusting for age (underlying time), OPV, OPV+VAS, OPV+MV, VAS, MV and year*age group.
* $p<0.05$.
VAS = vitamin A supplementation, MV = measles vaccine.

Supplementary Table S4. Mortality rates (per 100 person-years) and hazard ratios (HR) for after-campaign versus before-campaign for any campaign including oral polio vaccine (OPV) (i.e. also co-administered with vitamin A supplement (VAS) and measles vaccine (MV)), by cause of death. Analysis from 29 days to 35 months of age among children within the Chakaria HDSS from 2012 to 2019.

<u>**Respiratory infections**</u>

Campaign	Mortality rates per 100 person years (deaths / person years)		HR (After/Before-campaign) (95% CI) #1	Main model HR (After/Before-campaign) (95% CI) #2
	After-campaign	Before-campaign		
**29 days – 11 months**				
Any-campaign-OPV	0.34 (7/2074)	0.86 (103/11981)	0.55 (0.27-1.14)	0.56 (0.27-1.16)
Campaign-VAS-only	0.51 (15/2922)	0.85 (95/11133)	1.21 (0.65-2.27)	1.15 (0.62-2.13)
Campaign-MV-only	1.88 (1/53)	0.78 (109/14001)	8.53 (1.04-69.8)*	8.20 (1.01-66.3)*
**12 – 35 months**				
Any-campaign-OPV	0.08 (9/10613)	0.09 (15/16086)	0.71 (0.17-3.01)	0.71 (0.16-3.15)
Campaign-VAS-only	0.08 (17/20047)	0.11 (7/6652)	0.91 (0.35-2.37)	1.06 (0.44-2.54)
Campaign-MV-only	0.04 (2/4777)	0.10 (22/21922)	0.59 (0.13-2.61)	0.51 (0.11-2.31)
**Combined**				
Any-campaign-OPV	0.13 (16/12687)	0.42 (118/28067)	0.59 (0.31-1.12)	0.59 (0.31-1.15)
Campaign-VAS-only	0.14 (32/22968)	0.57 (102/17785)	1.14 (0.66-1.98)	1.08 (0.60-1.94)
Campaign-MV-only	0.06 (3/4830)	0.36 (131/35923)	0.94 (0.23-3.78)	0.84 (0.20-3.51)

#1: Adjusting for age (underlying time) and year*age group.
#2: Main multivariable model: adjusting for age (underlying time), any-OPV, VAS, MV and year*age group.
* $p<0.05$.

Other causes

Campaign	Mortality rates per 100 person years (deaths / person years)		HR (After/Before-campaign) (95% CI) #1	Main model HR (After/Before-campaign) (95% CI) #2
	After-campaign	Before-campaign		
**29 days – 11 months**				
Any-campaign-OPV	0.48 (10/2074)	0.49 (59/11981)	0.98 (0.46-2.09)	1.03 (0.48-2.20)
Campaign-VAS-only	0.55 (16/2922)	0.48 (53/11133)	1.65 (0.84-3.24)	1.65 (0.85-3.20)
Campaign-MV-only	0.00 (0/53)	0.49 (69/14001)	-	-
**12 – 35 months**				
Any-campaign-OPV	0.13 (14/10613)	0.12 (20/16086)	0.62 (0.20-1.86)	0.72 (0.23-2.30)
Campaign-VAS-only	0.13 (26/20047)	0.12 (8/6652)	1.03 (0.32-3.33)	0.98 (0.42-2.27)
Campaign-MV-only	0.13 (6/4777)	0.13 (28/21922)	1.68 (0.64-4.40)	1.48 (0.54-4.05)
**Combined**				
Any-campaign-OPV	0.19 (24/12687)	0.28 (79/28067)	0.84 (0.44-1.61)	0.92 (0.47-1.79)
Campaign-VAS-only	0.18 (42/22968)	0.34 (61/17785)	1.45 (0.78-2.68)	1.38 (0.73-2.61)
Campaign-MV-only	0.12 (6/4830)	0.27 (97/35923)	1.60 (0.63-4.08)	1.42 (0.53-3.77)

#1: Adjusting for age (underlying time) and year*age group.
#2: Main multivariable model: adjusting for age (underlying time), any-OPV, VAS, MV and year*age group.
* $p<0.05$.

Supplementary Figure S1. Schoenfeld residual plots for the main multivariable model for respiratory infections.

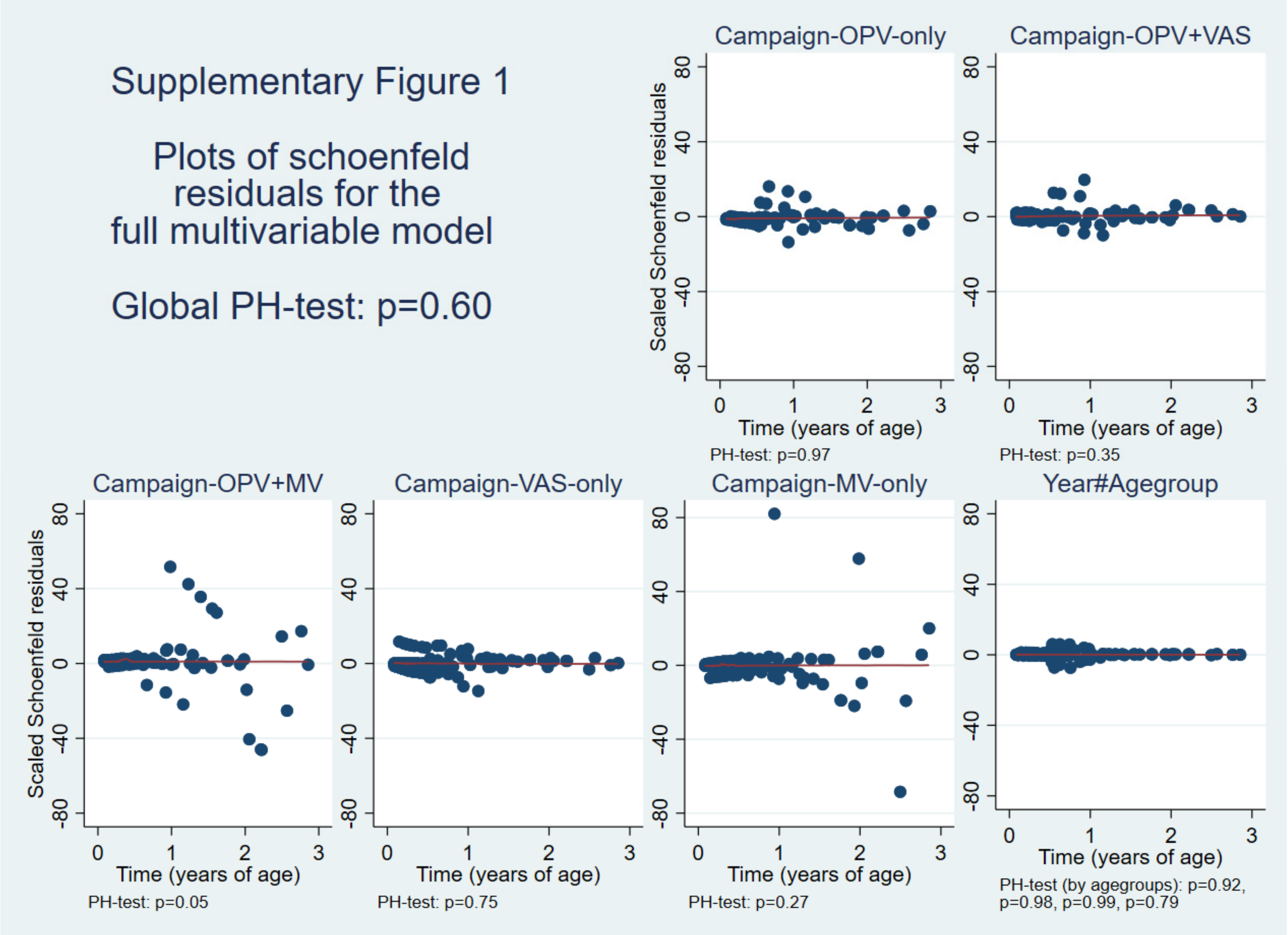

Supplementary Figure S2. Schoenfeld residual plots for the main multivariable model for other causes.

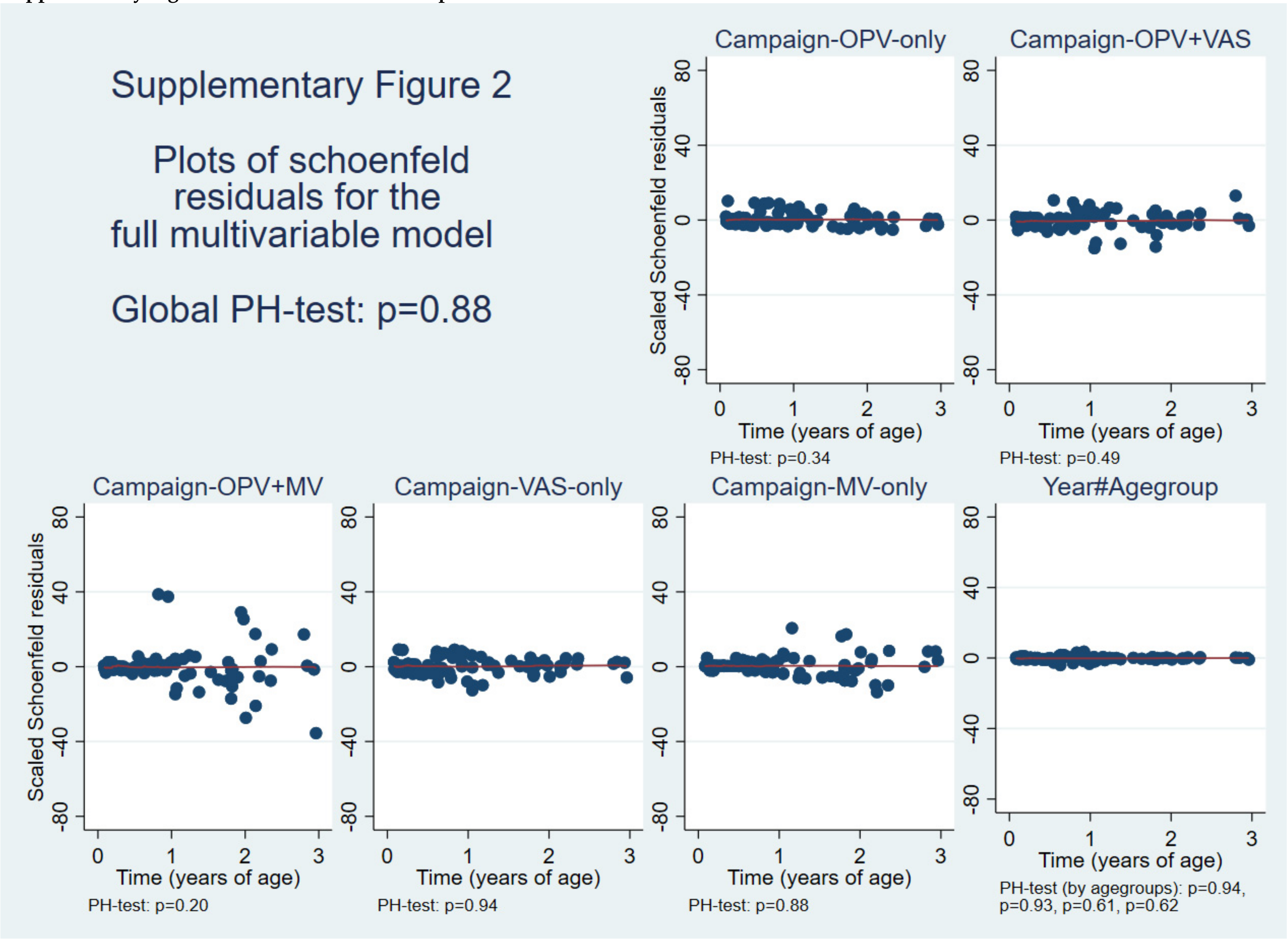

Supplement: Supplementary file 1 [file vaccines-09-01133-s001.zip › vaccines-1374424-supplementary.pdf]
